# An Ocular Protein Triad Can Classify Four Complex Retinal Diseases

**DOI:** 10.1038/srep41595

**Published:** 2017-01-27

**Authors:** J. J. W. Kuiper, L. Beretta, S. Nierkens, R. van Leeuwen, N. H. ten Dam-van Loon, J. Ossewaarde-van Norel, M. C. Bartels, J. D. F. de Groot-Mijnes, P. Schellekens, J. H. de Boer, T. R. D. J. Radstake

**Affiliations:** 1 Laboratory of Translational Immunology, department of Immunology, University Medical Center Utrecht, Utrecht, The Netherlands; 2 Department of Ophthalmology, University Medical Center Utrecht, Utrecht, The Netherlands; 3 Referral Center for Systemic Autoimmune Diseases, Fondazione IRCCS Ca’ Granda Ospedale Maggiore Policlinico di Milano, Milan, Italy; 4 Department of Ophthalmology, Deventer Hospital, The Netherlands; 5 Deparment of Medical Microbiology, University Medical Center Utrecht, Utrecht, The Netherlands; 6 Department of Clinical Immunology and Rheumatology, University Medical Center Utrecht, Utrecht, The Netherlands

## Abstract

Retinal diseases generally are vision-threatening conditions that warrant appropriate clinical decision-making which currently solely dependents upon extensive clinical screening by specialized ophthalmologists. In the era where molecular assessment has improved dramatically, we aimed at the identification of biomarkers in 175 ocular fluids to classify four archetypical ocular conditions affecting the retina (age-related macular degeneration, idiopathic non-infectious uveitis, primary vitreoretinal lymphoma, and rhegmatogenous retinal detachment) with one single test. Unsupervised clustering of ocular proteins revealed a classification strikingly similar to the clinical phenotypes of each disease group studied. We developed and independently validated a parsimonious model based merely on three proteins; interleukin (IL)-10, IL-21, and angiotensin converting enzyme (ACE) that could correctly classify patients with an overall accuracy, sensitivity and specificity of respectively, 86.7%, 79.4% and 92.5%. Here, we provide proof-of-concept for molecular profiling as a diagnostic aid for ophthalmologists in the care for patients with retinal conditions.

Complex diseases of the retina are responsible for approximately 20% of blindness in the developing world and cover a wide spectrum of vision-threatening conditions including intraocular inflammatory diseases (e.g. *uveitis*), retinal tearing or detachment, age-related macular degeneration, and potentially lethal and rare malignancies such as primary vitreoretinal lymphoma^1^. Optimal clinical management is key to preserving or restoring visual function, which is achieved by combinations of eye examination (e.g. fluorescein angiography), imaging technologies (e.g. optical coherence tomography) and functional tests. These clinical tools are effective, but bound by their requirement to be interpreted by well-experienced ophthalmologists to determine the optimal clinical care for each patient, which necessitate a high degree of training and experience^2^. The development of clinical decision tools based upon the molecular ‘fingerprint’ of patients could support the ophthalmologist in current evidence-based medicine and future personalized care. Aqueous humor of the anterior compartment of the eye provides a unique ‘liquid biopsy’ that could provide molecular information of the affected eye^3^.

We observed that small volumes of aqueous humor from representative common and complex retinal diseases display unique multi-protein profiles. We next investigated the potential of these profiles for its diagnostic power assessing a large cohort of four archetypical retinal diseases (*age-related macular degeneration, idiopathic non-infectious uveitis, primary vitreoretinal lymphoma,* and *rhegmatogenous retinal detachment*). Using multiple computational modeling strategies, we devised and validated a simple, but highly robust molecular classification model differentiating these ocular conditions in one single test. These findings form a proof-of-concept for ocular fluid-based decision tools and pave the way for the development of molecular workflows to guide personalized care in the near future.

## Results

### Exploratory analysis of the protein profile of AqH of retinal diseases

Aqueous humor paracentesis, the procedure for taking a small volume (<100 μl) of the acellular ocular fluid or aqueous humor (AqH) from the anterior chamber, is relatively safe and non-invasive in the hands of an experienced ophthalmologist^3^. AqH contains omnifarious protein contents that may be exploited to characterize the ocular microenvironment for diagnostic or scientific purposes^4^. To evaluate the potential of AqH proteins as classifiers for complex retinal diseases, we focused - as a proof-of-principle – on four clinically well-known retinal diseases that represent various facets of retinal pathology, including neoplasm (*primary vitreoretinal lymphoma* - PVRL), inflammation (*idiopathic non-infectious uveitis* - INIU), trauma (*rhegmatogenous retinal detachment* - RRD) and ‘degeneration’ (*age-related macular degeneration* - AMD). To maximize generalizability, we performed according to previous recommendation^5^, proteomic profiling of AqH of 175 patients using a discovery-based approach (n = 128) and an independent cohort (n = 47) to validate our findings. Demographics of the discovery and validation cohort are described in Table 1. The mean age of the discovery cohort was 66 years (range; 25–95), but significantly varied between the four disease groups, which is inherent to the representative age distribution for each of these retinal conditions (Table 1, Supplementary Table 1). In contrast, the mean age of the discovery and validation cohort was highly similar for each retinal disease (Table 1, Supplementary Table 1). The distribution of female and male patients was not significantly different between the groups of the discovery (*P* = 0.20) and replication cohort (*P* = 0.95, Table 1). Since there was a significant difference in age between the patients groups due to the age-related prevalence in the discovery cohort, we studied whether gender or age contributed to the observed differences in expression of protein levels. Multivariate analysis of covariance revealed no significant effect of age (F = 1.19, *P* = 0.26) or gender (F = 0.88, *P* = 0.62), thus, we proceeded to analyze the protein profiles of the AqH. We were able to detect the expression of 25 proteins in AqH (Supplementary Table 2). *Interferon-γ* and *basic Fibroblast growth factor* measurements were not detectable in the majority (>75%) of samples and also not specific for any disease group, thus, excluded for further analysis (see methods). The levels of most proteins in AqH were substantially different between the retinal disease groups studied (Supplementary Table 2 and Supplementary Figure 1).

### Cluster analysis reveals that retinal diseases are characterized by distinct intraocular protein signatures

**Figure 1:**
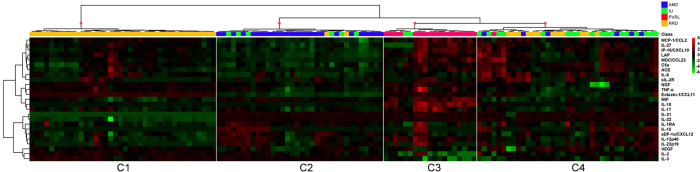
Heatmap of protein levels in ocular fluid of retinal diseases. Heatmap of unsupervised hierarchical clustering of the levels of 25 proteins in ocular fluid (aqueous humor) from patients with age-related macular degeneration (AMD in blue), primary vitreoretinal lymphoma (PVRL in red), idiopathic non-infectious uveitis (INIU in green), and rhegmatogenous retinal detachment (RRD in orange). Heatmap colors represent the fold changes from the overall mean concentration in a color-coded way: green, lower expression compared to the mean (zero); black, expression equal to the mean; red, higher expression compared to the mean. Dendrograms indicating the clustering relationships are shown to the left and above the heat map.

To reveal the underlying structure of the ocular microenvironment of retinal diseases, the AqH protein data were subjected to unsupervised hierarchical clustering to explore protein profiles that as a whole may be characteristic for each of the diseases. Global comparisons by hierarchical cluster analysis discerned 4 overarching groups labeled C1 to C4 with each distinct protein signatures (Fig. 1). The 4 clusters roughly corresponded with each of the 4 investigated retinal disease groups with RRD (C1) and PVRL (C3) being the most and AMD (C2) and INIU (C4) being the least homogeneous patient clusters (Fig. 1). C1 that exclusively contained RRD patients had relatively higher levels of Eotaxin-1/CCL11 and IL-3, with markedly low IL-21 and IL-22 levels compared to the other clusters. Cluster C3 represented the PVRL samples that contained high levels of IL-10, MIF, and low levels of IL-3. Eighty-one % of all AMD patients were clustered in C2, which was characterized by relatively lower levels of various proteins, including MCP-1/CCL2 and Eotaxin-1/CCL11. Most INIU samples (69%) were clustered in C4 and typically had higher levels of Angiotensin converting enzyme (ACE), Complement component 5a (C5a), IL-21, and IL-22 (Fig. 1). This analysis revealed that each of the investigated retinal diseases is characterized by distinct multi-protein profiles.

Note, 12/52 (23%) patients with RRD showed distinct overall AqH profiles within the RRD group that was characterized by relatively higher levels of IL-21, IL-22, and lower levels of Eotaxin, NGF, IL-2 and TNF-alpha (Supplementary Figure 2). We measured the same protein panel in paired vitreous fluid samples from the RRD patients of the discovery cohort, which revealed similar distinct mediator profiles in vitreous fluid (Supplementary Figure 2)

### Computational modeling reveals an intraocular protein signature that can be used to classify retinal diseases

**Figure 2:**
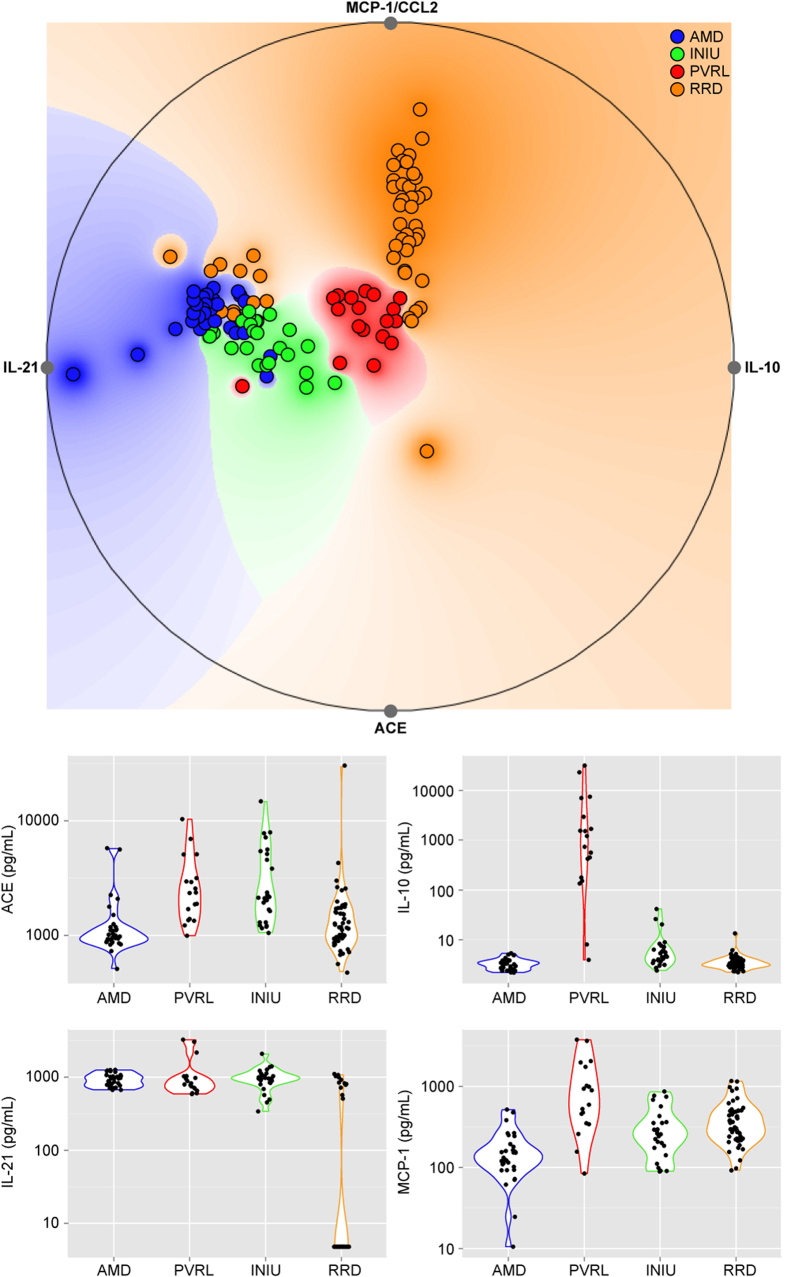
Projection-based multivariate visualization (RadViz^TM^) of the joint effect of four proteins in ocular fluid with the best capacity to correctly classify patients. The predictive accuracy to visually discriminate between the retinal diseases was equal to 79.6% on the basis of IL-10, IL-21, ACE, and MCP-1/CCL2 (VizRank method) is shown in the upper graph. Each point represents a patient that is colored according to the corresponding retinal disease. The levels of IL-10, IL-21, ACE, and MCP-1/CCL2 in aqueous humor for each of the retinal diseases are depicted in the bottom panel. AMD; Age-related macular degeneration, PVRL; Primary vitreoretinal lymphoma, INIU; Idiopathic non-infectious uveitis, RRD; Rhegmatogenous retinal detachment, IL; Interleukin, MCP-1/CCL2; Monocyte chemo-attractant protein-1, ACE; Angiotensin-converting enzyme.

**Figure 3:**
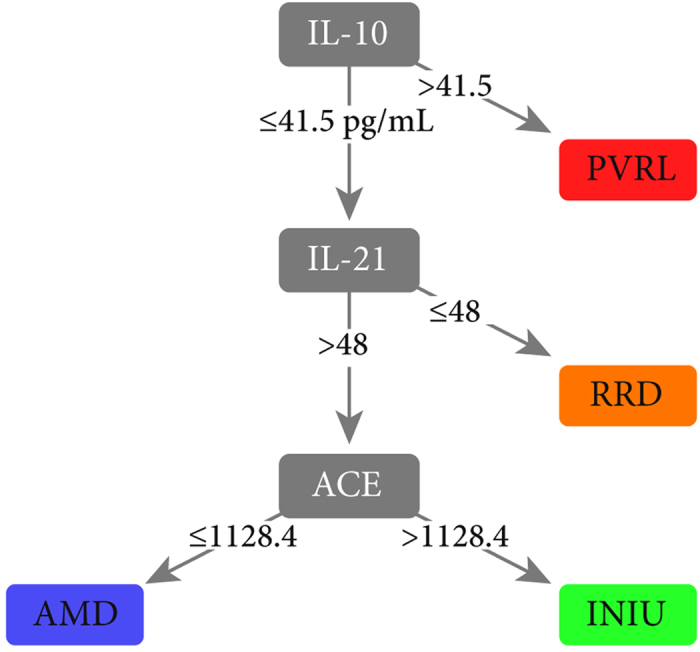
Decision algorithm that accurately distinguishes all four retinal diseases in a putative single measurement of ocular fluid (aqueous humor). The model was designed via the C4.5 decision tree classification algorithm in the training set. Independent replication case-series yielded and accuracy of 86.7%, sensitivity of 79.4% and specificity of 92.5%. The relative cut-off values are expressed in pg/mL. AMD; Age-related macular degeneration, PVRL; Primary vitreoretinal lymphoma, INIU; Idiopathic non-infectious uveitis, RRD; Rhegmatogenous retinal detachment, IL; Interleukin, ACE; Angiotensin-converting enzyme.

To provide a more understandable graphical interpretation of protein interactions, we used a projection-based visualization technique (Radviz^TM^), which provides a graphical representation of the samples ranked by the concerted effect of multiple proteins in an orthogonal space. Here, each sample is assigned a unique location within the circle as a function of its relative attraction to each of the proteins (higher or low expression levels) to explore meaningful patterns in AqH. On the basis of the joint-effect analysis via Radviz^TM^ the top projection identified, when considering four proteins (IL-21, IL-10, ACE, and MCP-1/CCL2) simultaneously, had a 79.6% accuracy to correctly classify patients indicating that separation can be obtained for four retinal diseases using simple protein-signatures (Fig. 2). Finally, we exploited the C4.5 algorithm, to produce a clinically useful decision algorithm that accurately distinguishes all four retinal diseases in a putative single measurement. The algorithm had a remarkable performance in the discovery case-series with an overall accuracy of approximately 84% that was robust against random variation in the dataset with a variance of nearly 1%, thus ensuring the putative reproducibility of data (Table 2). The final C4.5 tree model included just three proteins, IL-10, IL-21, and ACE (Fig. 3). This model and its relative cut-off values were determined in the training set. We subsequently measured the levels of IL-10, IL-21, and ACE in the replication cohort. When tested in the independent replication case-series, the three protein model yielded an accuracy of 86.7%, and a very good sensitivity (79.4%) and specificity (92.5%) (Table 3), underpinning the robustness of the model. In concordance with the discovery cohort, 2/13 RRD patients (15%) in the replication cohort also revealed relatively higher (>48 pg/mL) IL-21 levels compared to the other RRD patients.

## Discussion

In contrast to most other medical specialists, the ophthalmologist has the unique opportunity to visually examine the inside of the target organ – the eye. Not surprisingly, strategies to gather critical information for the management of retinal diseases are primarily based upon examination of the ocular fundus, usually supported by imaging technologies, functional, and laboratory tests^1^,^2^. Currently, clinical decision making relies profoundly on the interpretation of the ophthalmologist. In contrast, the coming era of *Personalized medicine* centers on the unique biological ‘fingerprint’ of patients to guide clinical decision making^6^. This ‘fingerprint’ can potentially be summarized by several key factors that serve as a proxy (biomarkers) for the detailed molecular make up of each patient that can be matched with their individual optimal care. Consequently, there is a pressing need for molecular tools and biomarkers to facilitate the realization of personalized medicine in ophthalmology^6^. While the last decade has seen an enormous rise of the adoption of molecular tools - mostly exploiting *next-generation sequencing* technologies - for rare monogenic inherited retinal conditions, objective molecular tools for more common and complex (i.e. multiple genes and environmental factors) retinal diseases are sparse^6^,^7^. Ideally, such molecular tools should be based upon objective biomarkers that can be measured directly from single samples such as ocular fluid or blood to have the potential to assist ophthalmologists in clinical decision-making.

Aqueous humor (AqH) is a continuously formed and dynamic ocular fluid that interchanges with many tissues inside the eye and is easily accessible via the anterior chamber of the eye by paracentesis^3^. The allure of AqH is that it can be considered a quick and minimally invasive ‘liquid biopsy’, representing a potentially rich source of ocular biomarkers such as proteins (e.g. cytokines), nucleotides and metabolites^4^. Curiously, AqH mediator levels commonly do not correspond well with the serum or plasma levels in patients^8^,^9^,^10^,^11^, which advocates the use of AqH complementary to blood in monitoring eye disease. The analysis of AqH in various retinal diseases has proved to be a useful tool for better understanding the complex underlying pathophysiology. Feasibility of analysis of AqH has also been facilitated by the rapid increase in availability of luminex-based multiplex technology that provides straightforward quantification of hundreds of biomarkers simultaneously in small volume samples^12^. Application of these multiplex-biomarker profiling assays also penetrated the field of ophthalmology including investigations of AqH in age-related macular degeneration^13^,^14^, uveitis^15^,^16^, vitreoretinal lymphoma^17^, and in retinal detachment^18^,^19^. Although this revealed aberrant expression of numerous ocular proteins in retinal diseases, AqH profiling strategies with the aim to support clinical management of multiple ocular conditions has mostly been neglected. Consequently, very few proteins from AqH are routinely being used in the clinic. An exception is the measurement of intraocular IL-10 and its ratio to IL-6, now considered a good biomarker for PVRL over uveitis^20^,^21^,^22^.

Complementary to AqH studies is the development of clinically useful protein profiling strategies of vitreous fluid (VF) such as a recent profiling to support diagnosis and treatment in a uveitis patient^23^. The relative distribution of individual proteins in AqH may not always reflect the levels found in VF^24^. Nevertheless, we previously demonstrated that the levels of IL-10 and IL-6 in AqH and VF in PVRL patients strongly correlate^20^. We also observed a good correlation between the levels of IL-21 (Spearman correlation coefficient r = 0.98, *P < *0.0001) and ACE (r = 0.40, *P = *0.003) in the paired AqH and VF samples from the 52 RRD patients of the discovery cohort. In fact, the overall protein profiles of AqH and VF revealed consistent molecular signatures (Supplementary Figure 2) underpinning the robustness of AqH profiling to monitor vitreoretinal diseases. Although like AqH the protein profiling of VF does provide an exciting basis for personalized medicine in the care of uveitis and PVRL^25^, paracentesis to obtain aqueous humor is easier, safer (side effects are rare), and less invasive than taking vitreous specimens^3^,^26^,^27^,^28^, making AqH-based approaches much more attractive to develop for a larger group of ocular conditions.

The aim of the study was to systematically test and provide proof-of-concept for AqH tests to be further explored as a potential tool for personalized medicine in ophthalmology, considering six criteria: (1) We included multiple complex ocular diseases simultaneously that should represent various facets of ocular pathology, (malignancy, inflammation, degeneration and trauma). (2) However, these disease groups should be generally well-distinguishable by ophthalmologists so we would be able to determine how well a putative AqH-algorithm would score. (3) We deliberately selected a panel of proteins that have been implicated in previous or related AqH studies^4^,^9^,^13^,^14^,^15^,^16^,^17^,^18^,^19^,^20^,^21^ and can be involved in more than one (e.g. IL-22, IL-6, or IL-17 are linked to AMD, Uveitis, and B cell lymphoma^29^,^30^,^31^,^32^) of these retinal conditions to challenge the discriminative power of the algorithm. (4) The panel of proteins was further selected based upon its availability within common multiplex platforms to facilitate that AqH biomarker assays do have the potential to become widely adopted. (5) Also, according to previous recommendations^5^ for executing discovery proteomic biomarkers studies, we restricted the number of proteins to augment statistical power and reduce false-positive outcomes. (6) We made use of a discovery-based approach and an independent replication cohort to validate the robustness of a potential model based upon AqH proteins. This strategy revealed to be fruitful since we robustly validated the high accuracy of the protein model in an independent cohort of these four diseases.

In this study, we illustrated two imperatives for the feasibility of molecular tools based on AqH. First, we reveal that AqH displays high resolution and unique protein profiles in a panel of diseases that provides a plethora of potential useful biomarkers. Secondly, intelligent data-visualization and decision tree analysis unmasked that these profiles can be reduced to selective biomarker panels, which support a straightforward and economical design for AqH-based classification tests. Therefore, as a *proof-of-concept*, we developed a simple modality for the four archetypical retinal diseases investigated. This model considered just three proteins simultaneously in a single measurement that performed with 84.1% overall accuracy, which we prospectively validated in an independent cohort with similar accuracy (86.7%). This demonstrated that parsimonious models may perform well in clinically feasible volumes of ocular fluid. In fact, we deliberately used only 25 μl of AqH, which is at least the available volume of the remainder of AqH routinely obtained for diagnostic purposes in suspected infection, neoplasm, or for the management of intraocular pressure.

As such, the results of this study pave the way for the development of AqH-based tools that could be applied to a wider variety of ocular diseases. In our opinion, such efforts should be tailor-made to specific clinical unmet needs, such as therapy response/resistance and early disease detection in longitudinal cohorts of patients. In this study we did not have power to stratify for all possible co-occurring diseases, however, the design made it possible to explore this concept briefly: 7/27 (26%) of PVRL cases in this study had a history or retinal detachment prior to PVRL diagnosis. Since PVRL cases may present with severe vitreous opacities preventing clear retinal examination, an accurate AqH test – such as the IL-10, IL-21, ACE triad – could discriminate between the differential diagnosis of INIU, PVRL and RRD (with AMD in this case serving as a negative control), contributing to early diagnosis and treatment thereby avoiding visual loss and improving prognosis.

In addition to being potent classifiers, IL-21, IL-10, and ACE may have distinct biological functions in these retinal diseases. IL-21 is a pleiotropic cytokine with diverse effects on a broad range of cell types including antigen-presenting cells (e.g. dendritic cells), T and B lymphocytes^33^. IL-21 signaling has strong pro-inflammatory capacities and plays a central role in the biology of uveitis^34^. IL-21 also induces proliferation of neoplastic B-cells in lymphoma^35^ and T helper 17 cell subsets, the latter cell subset involved in Uveitis and AMD^31^,^36^. Strikingly, a subgroup of RRD patients in the discovery and replication cohort (Supplementary Figure 2) revealed distinct overall AqH profiles that were characterized by proteins such as IL-21. It is tempting to speculate that this heterogeneity may be due to distinct molecular mechanisms or co-morbidities; The RRD cases in this subgroup with relatively higher IL-21 clustered together with the uveitis samples in Fig. 1. Curiously, chronic retinal detachment may have clinical inflammatory signatures, and vice versa, retinal detachment is a common complication in ocular inflammatory diseases^37^. In addition to its pro-inflammatory functions, however, IL-21 may also act as an immunosuppressive agent^33^ and thus, may either induce or suppress inflammation due to retinal damage in RRD. Although we could not link this subgroup to any clinically data (such as PVR development), probably due to the small size of this subgroup in our cohorts, this certainly provides an exciting field for further investigation and demonstrates the potential of AqH profiling in molecular classification of patients, which currently by ophthalmological examination alone were merely considered to be biologically homogenous.

PVRLs are mostly B cell malignancies^38^ notorious for their production of high levels of IL-10. Here, IL-10 is considered to function as an autocrine growth factor for malignant cells that also prevents apoptosis via *Bcl-2* induction, and has potent immunosuppressive capacities to favor tumor survival^38^,^39^.

*Angiotensin-converting enzyme* (ACE) has a pivotal role in the renin-angiotensin system and regulates blood pressure control, however, this enzyme also has important immunoregulatory functions^40^. ACE can be expressed by monocytic cells, such as epithelial macrophages^40^ and overexpression of ACE in macrophages results in increased pro-inflammatory cytokine (TNF-alpha, IL-6) production and an increase in the frequency of antigen-specific T cells in animal models^41^. Similar inflammatory signatures are implicated in the pathogenesis of non-infectious uveitis and support our observation of relatively increased levels of ACE in INIU compared to AMD. Since increased ACE in AqH has been reported for several ocular diseases^42^ – particularly sarcoidosis^43^– and ACE levels in AqH may be influenced by the use of medication affecting the renin-angiotensin system (ACE inhibitors), we used available clinical data to investigate this. (Supplementary Figure 3). None of the INIU patients in the discovery cohort had evidence or were suspected of sarcoidosis (follow-up ≥ 1 year) and the difference in intraocular ACE levels between INIU and AMD remained significant after correcting for ACE modulatory agents (Supplementary Figure 3).

We emphasize, however, that the here-investigated panel is illustrative and not exhaustive. The model’s predictive capability may be enhanced since it is limited by the fact that we investigated only a selective panel of proteins. Another limitation is that the current model assumes a *steady-state* of the levels of the proteins in AqH over time without measuring these at multiple time points in the same patients. Thus, it may be possible that other proteins, nucleotides or metabolites^44^ in AqH have great(er) potential to be used as biomarker in future studies. For example, small non-coding RNA such as microRNAs have been recently studied in AqH of patients with cataract and glaucoma^45^,^46^. Although promising biomarkers in other biofluids, the microRNA studies in (a relatively much larger amount of) AqH are currently exploratory and await more robust study design using discovery and validation cohorts between independent patients, which are critical to determine their diagnostic accuracy, robustness and potential over proteins.

The current study provides *proof-of-concept* in demonstrating that objective and pragmatic molecular tools for multiple ocular conditions simultaneously are now within reach, and raise the possibility for the development of clinical tools based upon the molecular architecture of the patient - or personalized medicine - in ophthalmology.

## Materials and Methods

### Patients

Aqueous humor (AqH) samples of 175 patients were collected at the outbound department of Ophthalmology at the University Medical Center Utrecht, The Netherlands. Patients included 128 subjects (discovery case-series) that underwent complete analysis of the AqH samples for 27 proteins that were used for computer modeling (see below). After design of the three protein model, an additional replication cohort of 47 subjects with each of the four disease groups was subsequently collected and used for a restricted analysis of proteins to validate prediction models (validation case-series). AqH samples were immediately stored at −80 °C after collection. Paired AqH and vitreous fluid samples from patients with rhegmatogenous retinal detachment (RRD) were collected during vitreoretinal surgery. AqH samples from patients with idiopathic non-infectious uveitis (INIU) or primary vitreoretinal lymphoma (PVRL) were the remainder of samples obtained with an anterior chamber paracentesis for diagnostic purposes. All included PVRL patients were immune-competent and had biopsy-proven diffuse large B cell lymphoma. The AqH samples from patients with neovascular age-related macular degeneration (AMD) were collected during paracentesis for lowering intraocular pressure prior to intravitreal anti-VEGF injections. Paracentesis was performed under the operation microscope in supine position. Patients with RRD, INIU and PVRL were not treated with any systemic or local immunomodulatory treatment at the time of sample collection. All participants gave their informed consent and the study was approved by the local medical ethics committee (University Medical Center Utrecht). All experimental protocols were carried out in accordance with the approved guidelines and were approved by the ethical committee of UMC Utrecht.

### Study Design

Twenty-seven proteins were simultaneously measured in 25 μl of undiluted AqH or 25 ul undiluted VF using our in-house developed^12^ and validated multiplex immunoassay based on *Luminex* technology (Supplementary Table 2). For statistical analysis, concentrations below the detection limit were converted to half of the lower limit of detection. When more than 25% of measurements were below detection limit, the mediator was excluded for further analysis, unless more than 90% was detected in one specific subgroup; overall 25 proteins respected all these prerequisites; all the concentrations are expressed in pg/mL throughout the paper.

### Statistical analysis

Conventional statistics analyses were conducted via the SPSS v22.0 software (IBM Corp, Armonk NY). To evaluate differences among groups, due to the non-normal distribution of variables in the studied groups, the Kruskal-Wallis test was used and results declared significant at the 0.002 level (α = 0.05/25) and subsequently the Dunn’s post-hoc test was used to assess the significance of pairwise comparisons at the 0.05 threshold (Supplementary Table 2). To categorize patients into groups with similar biological properties of the AqH, clustering methods were used. To this end, hierarchical clustering was performed on log-transformed data. Heatmaps were created based on the Pearson distance measure and the Ward’s linkage method using the MetaboAnalyst server^47^. Visualization of the joint effect of the log-transformed levels of proteins in AqH was performed using the radial coordinate visualization method Radviz^TM^ (Orange v2.7) to represent high dimensional data into the orthogonal space^48^. The best projection was evaluated (k-nearest neighbor classifier to evaluate the validity of the projection) via the VizRank method that scores 5,000 possible visualizations (jittering size; 0.1% of range) according to the degree of class separation^49^. Thus, proteins that harbor the most class information are most likely to be selected as a feature anchor on a unit circle in a Radviz^TM^ projection. A predictive clinical model was finally designed via the C4.5 decision tree classification algorithm^50^. To this end, the tree model was built on AqH data from the discovery set and internally validated after cross-validation; the final model was then tested in the external validation set to ensure the generalizability of data. Because the natural distribution of data may be suboptimal for learning classifiers in unbalanced datasets as it would overestimate the importance of the majority class^51^, the minority classes were oversampled via the synthetic minority oversample technique (SMOTE)^52^; each training set during validation was independently resampled and the testing set was never included into the resampling procedure and in data mining modelling, thus ensuring that the performance of the model is reproducible in unseen populations. Ten-fold cross-validation was used and the procedure repeated 100 times to estimate the variance of the classification tree; the performance of the final model was evaluated into the validation sets via contingency tables. The Weka 3.6.13 data mining software was used for the analysis^53^; the settings for the J48 algorithm (Weka implementation for C4.5) were: pre-pruning with a minimum of 15 instances per leaf and post-pruning with confidence factor = 0.01.

## Additional Information

**How to cite this article:** Kuiper, J. J. W. *et al*. An Ocular Protein Triad Can Classify Four Complex Retinal Diseases. *Sci. Rep.* **7**, 41595; doi: 10.1038/srep41595 (2017).

**Publisher's note:** Springer Nature remains neutral with regard to jurisdictional claims in published maps and institutional affiliations.

## Supplementary Material

Supplementary Information

## Figures and Tables

**Table 1:** Demographics of discovery and replication cohort investigated in this study.

Discovery Cohort	Total	AMD	PVRL	INIU	RRD
*N*	128	32	18	26	52
*Female/Male*	63/65	17/15	7/11	17/9	22/30
(*ratio*)	(1.0)	(1.1)	(0.6)	(1.9)	(0.7)
*Mean age (range) in years*	66 (25–95)	82 (66–95)	71 (52–82)	51 (25–79)	63 (40–85)
**Replication Cohort**					
*N*	47	7	9	18	13
*Female/Male*	22/25	4/3	4/5	8/10	6/7
(*ratio*)	(0.8)	(1.3)	(0.8)	(0.8)	(0.9)
*Mean age (range) in years*	66 (11–86)	82 (77–86)	69 (53–83)	51 (11–85)	64 (47–76)

AMD; age-related macular degeneration, PVRL; primary vitreoretinal lymphoma, INIU; idiopathic non-infectious uveitis, RRD; rhegmatogenous retinal detachment.

**Table 2:** Classification scores for the discovery cohort of the classification tree model.

	Sensitivity	Specificity	PPV	Balanced accuracy	AUROC
INIU	80.39% ± 6.4%	82.86% ± 1.23%	54.41% ± 2.57%	81.4 ± 3.75%	0.839 ± 0.195
AMD	71.28% ± 2.45%	87.48% ± 1.28%	65.57% ± 2.5%	79.4 ± 1.9%	0.863 ± 0.104
RRD	76.9% ± 0%	100% ± 0%	100% ± 0%	88.45 ± 0%	0.887 ± 0.197
PVRL	62.93% ± 20.11%	97.93% ± 1.04%	84.73% ± 7.18%	80.22 ± 10.61%	0.878 ± 0.291
**Model**	**75.15% ± 1.67%**	**93.09% ± 0.45%**	**75.13% ± 11.5%**	**84.12% ± 1.06%**	**0.87 ± 0.123**

INIU, idiopathic non-infectious uveitis; AMD, age-related macular degeneration; RRD, rhegmatogenous retinal detachment; PVRL, primary vitreoretinal lymphoma. PPV, positive predictive value; balanced accuracy, mathematical mean of sensitivity and specificity; AUROC, area under receiver operator characteristics. Values expressed as the mean ± standard deviation of 100 cross-validation runs.

**Table 3:** Performance of the 3 ocular protein classification tree in the validation case-series.

	Sensitivity	Specificity	PPV	Accuracy	Balanced accuracy
INIU	55.50%	93.10%	83.30%	78.70%	74.30%
AMD	85.70%	87.50%	54.50%	87.20%	86.60%
RRD	84.60%	85.29%	68.70%	78.72%	84.94%
PVRL	88.80%	100.00%	100.00%	97.87%	94.40%
**Model**	**79.40%**	**92.50%**	**80.35%**	**86.67%**	**85.95%**

See Table 2 for legend. Note that the model uses cut-off values defined during the discovery phases and thus AUROC values are not calculated.
