# Supplementary material for: An Ocular Protein Triad Can Classify Four Complex Retinal Diseases
Source: Sci Rep. 2017 Jan 27;7:41595. doi: 10.1038/srep41595 (PMC5269719; doi:10.1038/srep41595)
Supplement: Supplementary Information [file srep41595-s1.pdf]

Supplementary data for:

An Ocular Protein Triad Can Classify Four Complex Retinal Diseases

Authors: **J.J.W. Kuiper*[1,2], L. Beretta[3], S. Nierkens[1], R. van Leeuwen[2], N.H. ten Dam-van Loon[2], J. Ossewaarde-van Norel[2], M.C. Bartels[4], J.D.F. de Groot-Mijnes[2,5], P. Schellekens[2], J.H. de Boer†[1,2], T.R.D.J. Radstake†[1,6]**

Affiliations:

[1]Laboratory of Translational Immunology, department of Immunology, University Medical Center Utrecht, Utrecht, The Netherlands.

[2] Department of Ophthalmology, University Medical Center Utrecht, Utrecht, The Netherlands.

[3]Referral Center for Systemic Autoimmune Diseases, Fondazione IRCCS Ca' Granda Ospedale Maggiore Policlinico di Milano, Milan, Italy.

[4]Department of Ophthalmology, Deventer Hospital, The Netherlands.

[5]Deparment of Medical Microbiology, University Medical Center Utrecht, Utrecht, The Netherlands.

[6]Department of Clinical Immunology and Rheumatology, University Medical Center Utrecht, Utrecht, The Netherlands.

†These authors jointly directed this work.

*To whom correspondence should be addressed: Dr. Jonas Kuiper, Heidelberglaan 100, 3584CX, Utrecht, The Netherlands. 0031 (0) 88 755 1683, J.J.W.Kuiper@umcutrecht.nl.

**Age Discovery cohort**	***P* value[a]**	**Age Replication cohort**	***P* value[a]**	**Discovery vs Replication**	***P* value[a]**
AMD-PVRL	0.0021	AMD-PVRL	0.2520	AMD-AMD	0.99
AMD-INIU	< 0.0001	AMD-INIU	0.0005	PVRL-PVRL	0.99
AMD-RRD	< 0.0001	AMD-RRD	0.0930	INIU-INIU	0.99
PVRL-INIU	< 0.0001	PVRL-INIU	0.0332	RRD-RRD	0.99
PVRL-RRD	0.0113	PVRL-RRD	0.4765		
INIU-RRD	< 0.0001	INIU-RRD	0.0930		

Supplementary Table 1. Statistical comparison of the age of each disease group of the discovery and replication cohorts. AMD; age-related macular degeneration. PVRL; primary vitreoretinal lymphoma. INIU; idiopathic non-infectious uveitis. RRD; rhegmatogenous retinal detachment. [a] Holm-Šídák multiple comparison test.

Mediators	Total (n=128)	AMD (n=32)	PVRL (n=18)	INIU (=26)	RRD (n=52)	*P value All groups*[a]	*P value Pairwise Comparison*[b]					
	Median (range)	Median (range)	Median (range)	Median (range)	Median (range)		**AMD-PVRL**	**AMD-INIU**	**AMD-RRD**	**PVRL-INIU**	**PVRL-RRD**	**INIU-RRD**
**IL-1RA**	760.4(550.8-1387.7)	804.3(623.5-1114.8)	725.6(636.8-1387.7)	834.0(550.8-1351.7)	730.4(577.5-1238.1)	5.0E-04**	8.8E-01	1.0	4.0E-03*	6.0E-01	1.0	2.7E-03*
**IL-2**	8.2(1.0-36.1)	7.2(3.6-14.4)	2.1(1.0-25.7)	8.8(1.3-36.1)	8.9(3.1-15.5)	2.1E-04**	3.7E-01	7.3E-01	6.6E-02	1.1E-02*	2.5E-04*	1.0
**IL-3**	2452.3(406.0-3612.2)	2576.4(1055.8-2990.6)	1523.4(406.0-2245.9)	1974.5(888.7-3299.5)	2688.8(1614.7-3612.2)	1.2E-10**	5.8E-06	1.5E-02*	1.0	2.1E-01	2.5E-09*	5.9E-05*
**IL-6**	96.0(10.6-21446.9)	37.9(10.6-17188.6)	62.9(18.1-334.5)	116.7(13.4-21446.9)	165.6(18.3-10587.2)	2.0E-03						
**IL-10**	3.6(2.2-31646.0)	3.3(2.2-5.3)	975.5(3.9-31646.0)	4.7(2.4-41.5)	3.3(2.2-13.4)	1.4E-12**	1.6E-10*	1.2E-03*	1.0	8.2E-03*	2.8E-10*	4.0E-03*
**IL-12p70**	474.7(348.7-754.2)	481.1(348.7-623.2)	541.8(449.9-754.2)	521.2(407.3-629.2)	445.8(379.3-720.6)	1.4E-09**	3.8E-02	9.9E-01	3.5E-03*	9.2E-01	4.7E-08*	1.2E-05*
**IL-15**	0.9(0.9-342.2)	0.9(0.9-63.7)	0.9(0.9-342.2)	10.8(0.9-157.2)	0.9(0.9-4.9)	7.1E-08**	1.0	1.0	1.1E-02*	1.2E-01	1.0	8.7E-05*
**IL-17**	49.5(25.3-182.1)	45.8(25.3-60.3)	71.5(38.0-182.1)	58.5(36.9-81.1)	46.2(34.2-80.3)	1.3E-07**	1.2E-05*	7.4E-04*	1.0	1.0	4.5E-05*	3.0E-03*
**IL-21**	780.7(4.8-3245.8)	917.5(667.7-1257.8)	807.9(585.6-3245.8)	955.1(339.7-2081.5)	4.8(4.8-1103.3)	2.2E-13**	1.0	1.0	3.6E-09*	1.0	4.3E-05*	3.3E-09*
**IL-22**	387.4(1.1-562.1)	395.9(195.3-502.1)	438.3(377.7-556.2)	426.6(326.5-560.5)	104.7(1.1-562.1)	9.8E-15**	5.8E-02	3.4E-01	1.8E-05*	1.0	2.1E-10*	6.1E-10*
**IL-23**	16.4(3.4-844.7)	34.2(3.4-128.3)	11.6(8.9-844.7)	64.0(8.9-168.7)	8.9(3.6-167.7)	1.5E-07**	1.0	5.4E-01	1.5E-03*	3.1E-01	8.1E-02	7.0E-07*
**IL-27**	27.6(1.0-4082.6)	11.3(1.0-445.0)	124.4(1.0-4082.6)	55.7(1.0-826.3)	33.2(1.0-837.3)	3.7E-06**	3.4E-05*	1.2E-04*	3.2E-03*	1.0	2.5E-01	8.8E-01
**TNF-α**	8.2(3.8-39.3)	7.2(3.8-10.1)	8.7(6.5-39.3)	8.0(6.2-11.1)	8.6(5.7-16.3)	1.3E-03						
**MIF**	6757.2(206.8-226077.4)	7816.8(1585.7-30051.8)	51534.0(596.5-226077.4)	8004.3(813.8-22139.8)	3393.3(206.8-20630.4)	2.7E-09**	2.4E-02*	1.0	7.9E-04*	1.3E-03*	2.7E-09*	1.1E-01
**LAP**	3520.6(2157.7-11553.8)	2773.2(2157.7-7804.0)	4362.5(3526.5-10128.9)	3371.2(2219.6-11553.8)	3607.9(2323.5-6110.5)	3.3E-09**	2.6E-09*	9.4E-03*	2.8E-05*	6.4E-03*	1.9E-02*	1.0
**MCP-1/CCL2**	273.2(10.6-3768.0)	142.0(10.6-518.2)	744.5(84.3-3768.0)	260.0(89.1-865.5)	308.4(92.1-1166.0)	2.4E-09**	1.5E-08*	2.7E-02*	1.6E-06*	6.0E-03*	1.7E-01	5.4E-01
**Eotaxin-1/CCL11**	27.4(8.8-72.1)	13.4(8.8-33.7)	31.8(26.3-42.6)	16.8(10.5-72.1)	39.0(10.1-50.1)	1.7E-12**	2.3E-05*	1.3E-01	4.1E-12*	8.4E-02	1.0	1.7E-04*
**MDC/CCL22**	14.7(6.5-734.0)	10.6(6.5-70.7)	46.4(11.1-414.5)	15.6(8.2-734.0)	15.1(7.7-324.7)	2.8E-08**	1.7E-08*	6.1E-04*	2.5E-03*	1.1E-01	2.7E-03*	1.0
**IP-10/CXCL10**	174.4(6.6-17375.1)	67.2(6.6-961.8)	1629.7(137.5-17375.1)	478.6(31.3-16912.5)	170.4(44.4-1146.2)	2.7E-10**	5.0E-10*	2.0E-05*	3.8E-03*	1.5E-01	1.7E-04*	3.4E-01
**SDF-1α/CXCL12**	1152.2(773.1-1400.9)	1210.1(953.7-1400.9)	1115.2(988.8-1363.0)	1190.0(905.4-1342.1)	1099.3(773.1-1385.3)	1.1E-04**	2.0E-01	1.0	2.9E-04*	5.6E-01	1.0	5.3E-03*
**NGF**	1.9(0.0-7.4)	1.6(0.7-2.9)	3.2(1.6-7.4)	1.7(0.0-2.7)	2.3(0.1-4.1)	9.3E-10**	4.3E-09*	1.0	6.5E-04*	1.5E-06*	3.3E-03*	4.9E-02*
**VEGF-A**	276.8(0.7-2638.7)	93.9(21.6-2638.7)	215.8(28.8-1100.8)	548.2(27.3-1863.9)	323.0(0.7-1098.3)	2.0E-04**	1.0	7.3E-04*	4.0E-02*	5.8E-03*	1.6E-01	5.5E-01
**sIL-2R**	888.4(396.2-37985.9)	884.2(625.6-11880.0)	1525.7(632.2-11206.6)	1143.2(396.2-11203.9)	754.6(566.6-37985.9)	7.4E-03						
**C5a**	24.9(0.1-766.8)	4.6(0.1-407.4)	58.6(1.2-766.8)	90.2(0.1-642.9)	21.2(0.2-554.2)	2.5E-06**	2.3E-03*	6.4E-06*	3.0E-01	1.0	1.6E-01	2.4E-03*
**ACE**	1288.8(473.0-30396.0)	986.9(510.1-5775.5)	2355.4(993.4-10357.2)	2149.5(1049.9-14819.2)	1170.6(473.0-30396.0)	7.6E-09**	3.0E-05*	2.7E-06*	1.0	1.0	5.9E-04*	7.0E-05*

Supplementary Table 2. Mediator concentrations in aqueous humor of the discovery cohort. AMD; Age-related macular degeneration, PVRL; Primary vitreoretinal lymphoma, INIU; Idiopathic non-infectious uveitis, RRD; Rhegmatogenous retinal detachment, IL; Interleukin, MIF; Macrophage migration inhibitory factor, LAP; latency-associated protein of transforming growth factor-beta 1, MCP/CCL2; Monocyte chemo-attractant protein-1, MDC/CCL22; Macrophage-derived chemokine, IP-10/CXCL10; Interferon gamma-induced protein 10, SDF-1α/CXCL12; Stromal cell-derived factor 1, NGF; Nerve growth factor, VEGF; Vascular endothelial growth factor, sIL-2R; Soluble IL-2 receptor, C5a; Complement component 5a, ACE; Angiotensin-converting enzyme. [a] Kruskal-Wallis test.[b] Dunn's post-hoc test. ***P* <0.002; **P* <0.05

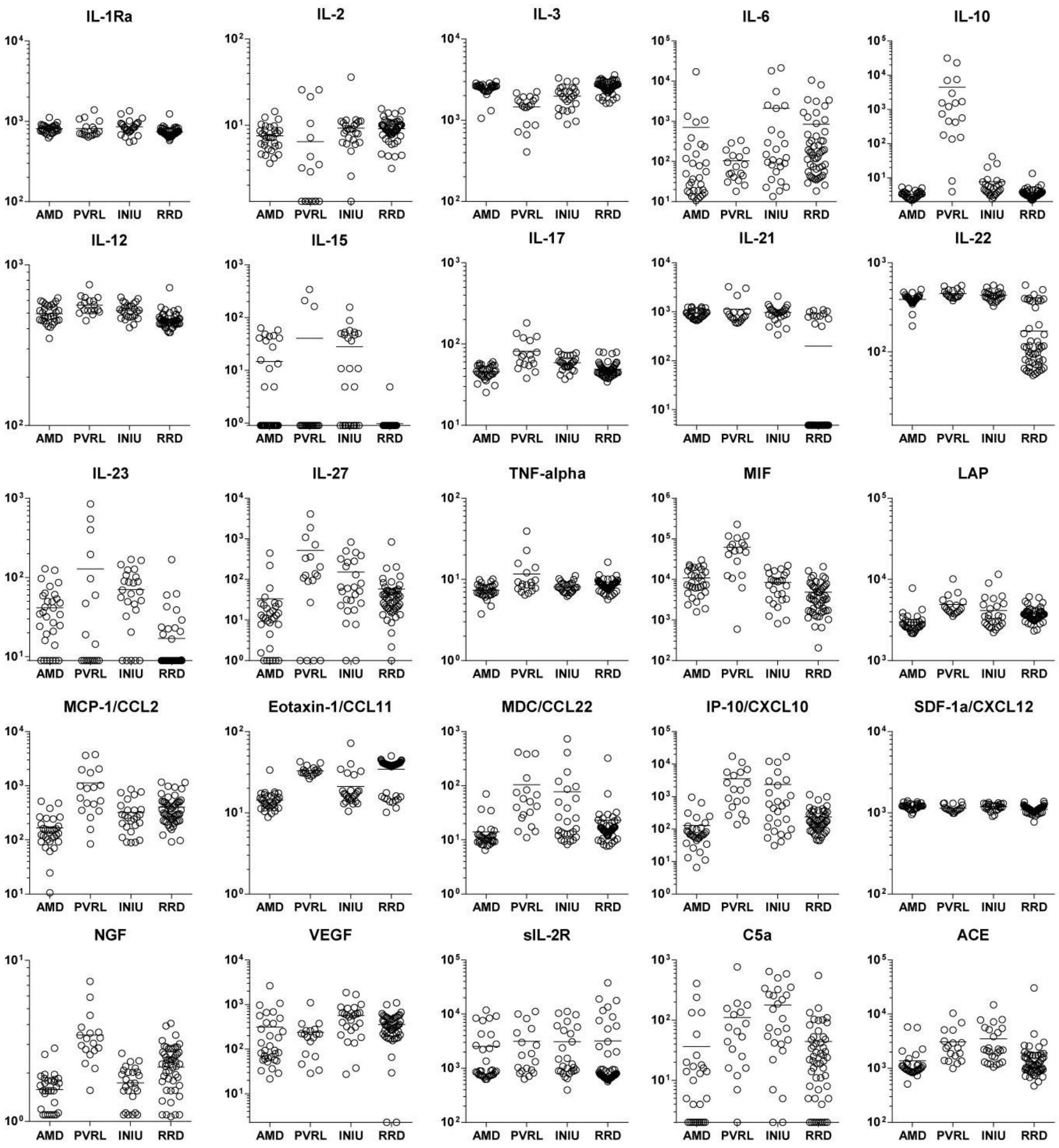

Supplementary Figure 1. Scatterplots of the mediator concentrations in aqueous humor of the discovery cohort. AMD; Age-related macular degeneration, PVRL; Primary vitreoretinal lymphoma, INIU; Idiopathic non-infectious uveitis, RRD; Rhegmatogenous retinal detachment, IL; Interleukin, MIF; Macrophage migration inhibitory factor, LAP; latency-associated protein of transforming growth factor-beta 1, MCP/CCL2; Monocyte chemo-attractant protein-1, MDC/CCL22; Macrophage-derived chemokine, IP-10/CXCL10; Interferon gamma-induced protein 10, SDF-1α/CXCL12; Stromal cell-derived factor 1, NGF; Nerve growth factor, VEGF; Vascular endothelial growth factor, sIL-2R; Soluble IL-2 receptor, C5a; Complement component 5a, ACE; Angiotensin-converting enzyme. For statistical analysis see supplementary table 2.

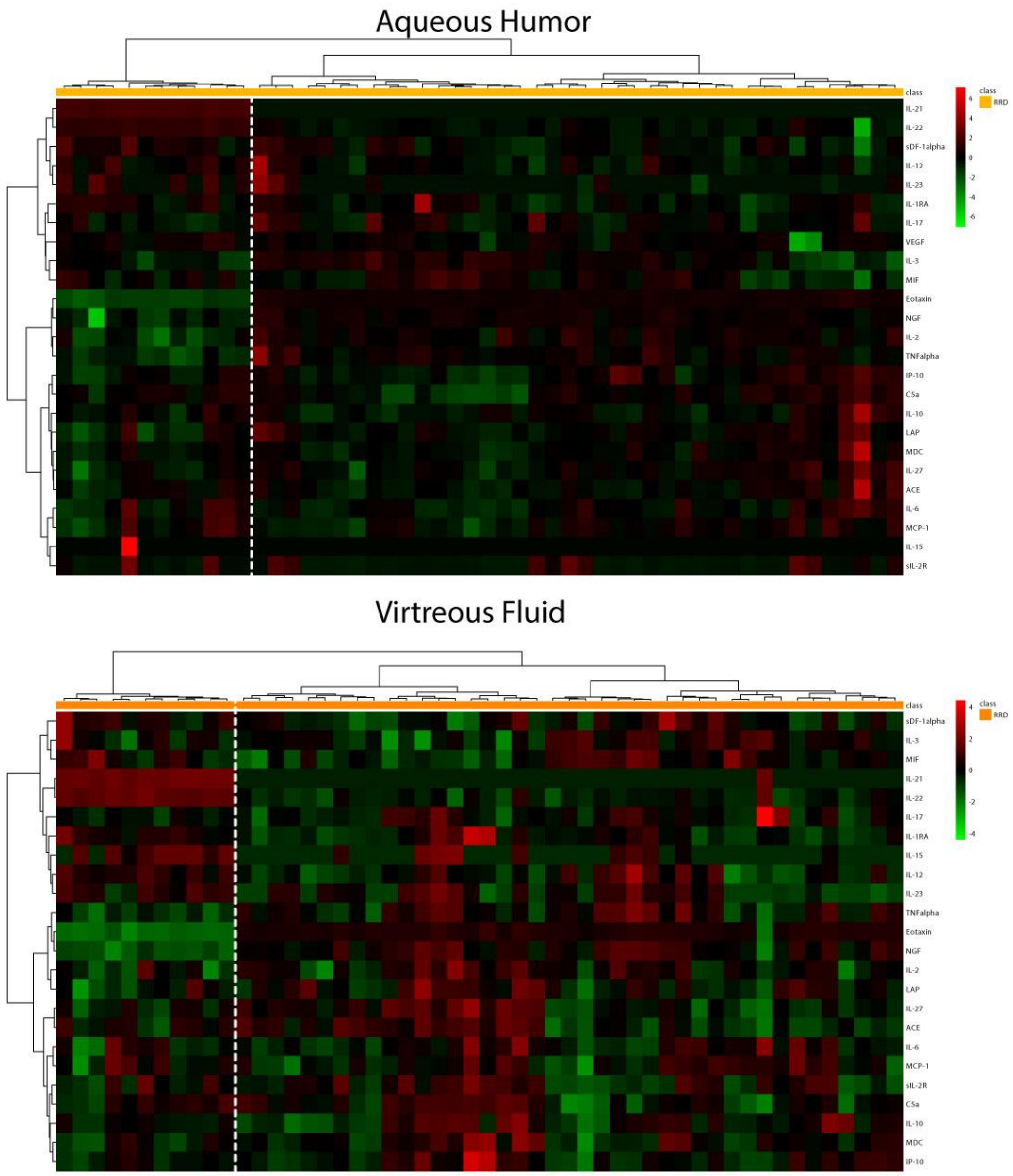

Supplementary Figure 2. Heatmap of protein levels in paired ocular fluids of patients with rhegmatogenous retinal detachment (n=52). Heatmap of unsupervised hierarchical clustering of the levels of 25 proteins in paired aqueous humor and vitreous fluid from patients with rhegmatogenous retinal detachment (RRD in orange) from the discovery cohort. Heatmaps were created based on the Pearson distance measure and the Ward's linkage method using the MetaboAnalyst server[47]. Heatmap colors display the fold changes from the overall mean concentration: green, lower expression compared to the mean; black, expression equal to the mean; red, higher expression compared to the mean. Dendrograms indicating the clustering relationship are shown to the left and above the heat map. White dotted line indicates the subgroup of patients within the RRD disease group as described in the manuscript. VEGF measurements were not detectable in the majority of vitreous samples and excluded for analysis

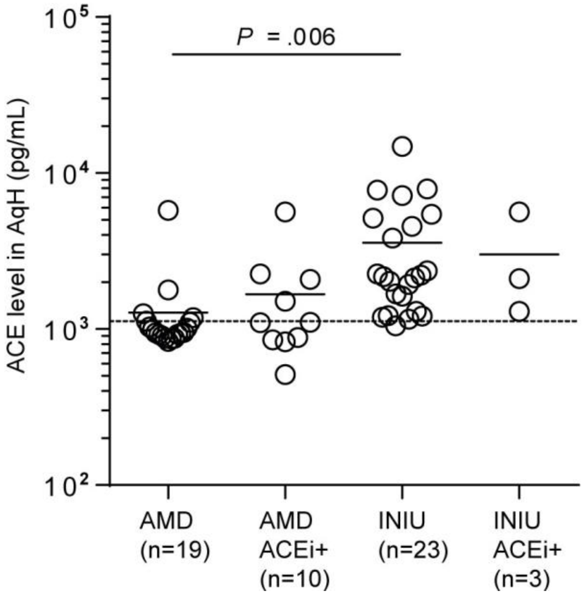

Supplementary Figure 3. Scatterplot of the levels of Angiotensin-converting enzyme in aqueous humor of the patients with Idiopathic non-infectious uveitis (INIU) and Age-related macular degeneration (AMD) of the discovery cohort. Patients were subdivided according to use of medication targeting the renin-angiotensin system (ACEi+ = current or recent history of *perindopril*, *acupril*, *metoprolol*, *irbesartan*, *captopril*, *atacand*, *hydrochlorothiazide*/ *losartan*). Data were available for 29/32 AMD patients and 26/26 INIU patients. Dotted line indicates the cut-off value of 1128.4pg/mL from Figure 3. Mean difference between AMD and INIU patients was tested with unpaired T-test (two-tailed).
